# Prevalence of left ventricular systolic dysfunction measured by *Global longitudinal* strain echocardiography in chronic kidney disease patients with preserved left ventricular ejection fraction

**DOI:** 10.1016/j.ihj.2025.12.003

**Published:** 2025-12-11

**Authors:** Muneshwar Kumar, Satyajit Singh, Vinay Singh Rathore, Surendra Kumar Naik, Abhishek Kumar, Chandra Prakash Thakur, Amratansh Varshney

**Affiliations:** a Department of Cardiology, AIIMS Raipur, C.G., India; b Department of Nephrology, AIIMS Raipur, C.G., India

**Keywords:** GLS, CKD, Preserved LVEF

## Abstract

**Objective:**

To study the prevalence of LV systolic dysfunction measured using GLS in patients of CKD with normal LVEF and correlation of GLS with the biochemical parameters and to determine the incidence of all-cause mortality and its correlation with GLS.

**Patients and methods:**

This study included 100 CKD patients of all stages, enrolled from July 2022 to June 2023. Global Longitudinal Strain (GLS) was calculated using speckle tracking echocardiography following relevant investigations. Patients were followed for one year to determine the incidence of all-cause mortality and its correlation with GLS.

**Results:**

Males were the predominant gender (66 %). The mean age of patients was 52yrs (±13.59). Maximum patients (47 %) were in ESRD stage (stage 5 CKD), followed by stage 4 CKD (29 %). Abnormal GLS (>-16 %) was prevalent in 98 % of study population. Mean GLS was −11 % ± 2.74. Patients with LVGLS ≥ -11 % were more likely to have higher prevalence of co-morbidities. Statin use was more prevalent in those patients with LVGLS ≥ -11 % (*p* = 0.029). In the median follow up of 1-year all-cause mortality was 22 % (*n* = 21), however there was no significant correlation of mortality with GLS.

**Conclusion:**

Patients with abnormal LVGLS (≥-11 %) had more comorbidities. Abnormal global longitudinal strain (GLS > −16 %) was prevalent in 98 % of the study population. The higher prevalence compared to other studies may be related to the lesser use of nephroprotective and cardioprotective drugs (e.g., ACEI/ARB in only 28 %); however, abnormal GLS was not correlated with mortality.

## 1 Introduction

The prevalence of CKD has been reported in an increasing number of studies worldwide. Chronic kidney disease is a progressive condition that affects >10 % of the general population worldwide, amounting to >800 million individuals^1^ and the pooled prevalence of CKD from community-based studies in India is 13.24 %.^2^ Reasons for the increasing incidence and prevalence of advanced CKD, among others, include aging populations, increasing prevalence of type 2 diabetes and hypertension, and a low detection rate and therapeutic inertia in the early stages of CKD. In 25- to 34-year-old patients with end-stage kidney disease, annual mortality is increased 500- to 1000-fold and corresponds to that of the ≈85-year-old general population.^3^ Ischemic heart disease, which causes myocardial infarction and angina, is one of the cardiovascular manifestations of CKD, other manifestations of CKD are hypertrophy of the left ventricle, LV diastolic and systolic dysfunction and dilated cardiomyopathy. It is possible that microvascular ischemia contributes to the development of uremic cardiomyopathy, as the hypertrophy of the LV is connected to a decrease in capillary density, particularly in the central endocardium.

Although EF is most commonly used parameter for the assessment of LV systolic function, it has many limitations including its inability to diagnose and risk stratify patients with heart failure with preserved EF. HFpEF is prevalent in CKD patients. LVEF has poor sensitivity in detecting early myocardial impairment. Increasing evidence on global left ventricular longitudinal strain (GLS) suggests superiority over left ventricular ejection fraction (LVEF) in risk stratification.^4^ In healthy individuals GLS range from −16 to −19 %. A cut off at −16 % has been shown to provide important risk stratification and prognostic value. Therefore **Krishnasamy et al** defined impaired GLS as > −16 % (a less negative value reflects a more impaired GLS).^5^

GLS has been proposed as the test of choice in guidelines for monitoring of asymptomatic cardiotoxicity related to chemotherapy. It also has the potential to improve risk stratification, redefine criteria for disease classification, and determine treatment in asymptomatic LVD resulting from a variety of etiologies.^6^ However, there is paucity of studies evaluating Prevalence of left ventricular systolic dysfunction measured by *Global longitudinal s*train echocardiography in chronic kidney disease patients with preserved left ventricular ejection fraction.^7^

## 2 Methodology

This single centered, observational, prospective cohort study included 100 CKD aged more than 18 years of age visiting Department of Nephrology and Cardiology from July 2022 to June 2023. Patients were followed up for 1 year from the date of enrollment for determining the prevalence of all-cause mortality and correlation with GLS. Patients with LVEF *<*50 %, Atrial fibrillation, Previous MI, Valvular heart diseases, those having history of coronary interventions were excluded.

Demographic, Clinical and Laboratory data of patients were recorded in a proforma by interviewing patient and reviewing medical records. CKD was defined as abnormalities of kidney structure or function, present for >3 months, with implications for health," and requires one of two criteria documented or inferred for >3 months: either GFR <60 mL/min/1.73 m2 or markers of kidney damage, including albuminuria.^8^ Estimated glomerular filtration rate (eGFR) was calculated by the CKD Epidemiology Collaboration (CKD-EPI) equation. Hypertension was defined as BP ≥ 140/90 mmHg. Diabetes mellitus was defined as FBS >126 mg/dl or RBS >200 mg/dl or HbA1C >6.5 %. Dyslipidemia was defined as LDL >130 mg/dl or TG > 200 mg/dl. Obesity was defined was BMI ≥25 kg/m2.

**Figure:**
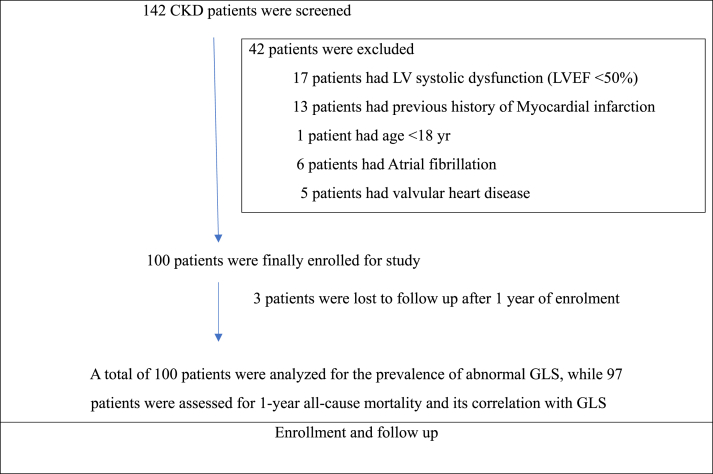

**Fig. 1:**
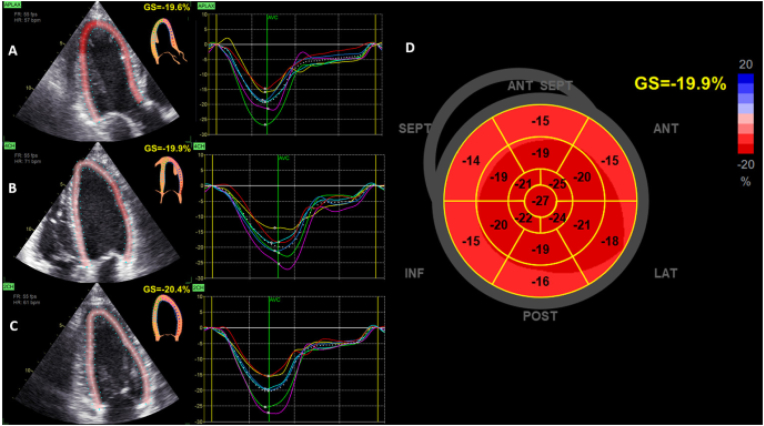
Left ventricular GLS assessment with two-dimensional speckle tracking echocardiography. The figure demonstrates analysis of left ventricular GLS from the three-chamber (A), four-chamber (4CH) (B) and two-chamber (2CH) (C) views, with the respective time to strain curves. The value of GLS is displayed in each view. (D) Polar map with the regional values and the GLS value calculated from the 17 segments which is within the normal value.

Transthoracic echocardiography was performed using Phillips cardiac ultrasound system (EPIQ 7C). All the echocardiographies were done by the same investigator to avoid interobserver variability. Each study was performed with subject in the left lateral decubitus position using standard apical four chamber, three chamber and two chamber views according to the recommendations of the American Society of Echocardiography. Left ventricular systolic function was calculated by Simpson's formula and LV GLS was calculated using two-dimensional speckle tracking echocardiography images of apical four, three and two chamber view. Left ventricular GLS was provided by the software as the average peak systolic longitudinal strain value of the three views (Fig. 1).

## 3 Statistical analysis

Statistical analysis was done using STATA 14.2 (updated version 2018). Quantitative data was analyzed using Mean, Standard Deviation (SD) and *t* test. Correlation among different variables were analyzed by Pearson's correlation coefficient. Qualitative data was analyzed using Chi Square Test, One way ANOVA, MANOVA and Fisher's test or appropriate test as necessary. Difference between two variables were considered significant when ‘p’ value is less than 0.05.

## 4 Results and observations

This study included 100 patients with chronic kidney disease (CKD). The mean age was 52 years (±13.59), 66 % (*n* = 66) were males, 47 % (*n* = 47) had diabetes, 81 % (*n* = 81) had hypertension and 34 % (*n* = 34) had dyslipidemia. The largest group of patients (47 %, *n* = 47) was in ESRD (stage 5 CKD), followed by stage 4 CKD (29 %, *n* = 29), and stage 3b CKD (15 %, *n* = 15) (Table 1). 53 % (*n* = 53) patients were on dialysis therapy. Of those 92 % (*n* = 48) were receiving haemodialysis and 8 % (*n* = 5) were receiving peritoneal dialysis. The mean chronic kidney disease (CKD) duration was 32.7 (SD: 35.7) months, mean eGFR was 22.52 (SD: 21.94) mL/min/m^2^, mean creatinine was 5.10 (SD: 3.59) mg/dL, mean albumin was 3.43 (SD: 0.68) g/dL, and mean urinary ACR was 2472.09 (SD: 3325.61) mg/g. Among patients, 67 % (*n* = 67) were taking diuretics. The most frequently used antihypertensive was the calcium channel blocker (CCB) at 66 % (*n* = 66), followed by the beta blocker at 30 % (*n* = 30) and ACEI/ARB at 26 % (*n* = 26). Regarding other therapies, statin use was observed in 39 % (*n* = 39) of subjects, erythropoietin in 52 % (*n* = 52), and sodium bicarbonate supplementation in 71 % (*n* = 71). Normal LVEF (average 66.61 % ± 8.13), as measured by Simpson's method, was observed in all patients. Average e/lateral e’ was 11.59 ± 5.15, while average e/medial e’ was 13.92 ± 6.5. Left ventricular hypertrophy (LVH) was present in 61 % (*n* = 61) of patients. Abnormal GLS (less than −16 %) was prevalent in 98 % (*n* = 98) of the study population, with an average GLS of −11.01 % ± 2.74.

**Table 1:** Summary of GLS by eGFR Category.

eGFR (ml/min/1.73m^2^)	Summary of GLS			*P* value between groups
	Mean	Std deviation	frequency	
<15	−11.6 %	2.4	47	0.06
15–29	−9.7 %	2.9	29	
30–44	−11.5 %	2.9	15	
45–59	−12.8 %	1.2	2	
60–89	−10 %	0.8	3	
>90	−11.45 %	2.7	4	

There was no statistically significant difference in GLS value between the groups (*p* = 0.6)

Patients with LVGLS ≥ -11 % had a higher prevalence of co-morbidities like diabetes (*p* = 0.10), hypertension (*p* = 0.17) and obesity (*p* = 0.12), though not clinically significant.

Statin use was significantly more prevalent among patients with LVGLS > -11 % (*p* = 0.029). Patients with LVGLS ≥ -11 % had relatively lower LVEF (*p* = 0.07). There was no significant difference in LVH, diastolic dysfunction, or mortality between the two groups (Table 2).

**Table 2:** **Characteristics of patients with preserved left ventricular (LV) ejection fraction and LV global longitudinal strain (GLS)** ≥ -**11 % vs. LV GLS *<****-***11 %**.

Variables	LVGLS > - 11 % (*n* = 51)	LV GLS < -11 % (*n* = 49)	*P* value
Age (years)	53 (+12.6)	51 (+14.5)	0.39
Male gender	36 (71 %)	30 (61 %)	0.32
Hypertension	44 (86 %)	37 (75 %)	0.17
Diabetes Mellitus	28 (55 %)	19 (39 %)	0.10
Obesity	11 (22 %)	5 (11 %)	0.12
Hypercholesterolemia	17 (33 %)	17 (35 %)	0.88
Medications			
ACEI/ARB	14 (28 %)	12 (25 %)	0.69
BB	14 (27 %)	16 (32 %)	0.57
Statin	25 (50 %)	14 (28 %)	**0.029**
Diuretics	33 (64 %)	34 (69 %)	0.62
CCB	35 (68 %)	31 (63 %)	0.57
Phosphate binder	5 (10 %)	7 (14 %)	0.49
K binder	6 (12 %)	10 (20 %)	0.29
erythropoietin	25 (49 %)	27 (55 %)	0.54
NaHCO3	36 (70 %)	35 (71 %)	0.96
LVEF	65.1 (±8.4)	68.1 (±7.7)	**0.07**
LVH (%)	31	30	0.964
E/Lateral e’	11.9 (±5.4)	11.27 (±4.9)	0.54
expired	11 (21.5 %)	10 (20.4 %)	0.79

Pearson's pairwise correlations showed that GLS was negatively correlated with diabetes mellitus (*p* = 0.05), statin requirement (*p* = 0.05), serum sodium level (*p* = 0.07), serum sodium bicarbonate level (*p* = 0.02), and pH (*p* = 0.09), and was positively correlated with phosphate (*p* = 0.01) (Table 3)

**Table 3:** Pearson's pairwise correlations of GLS with other variables.

Variables	Pearson correlation coefficient (*r)*	Significance value
Age (years)	−0.09	0.36
Female	0.10	0.28
Male	−0.11	0.27
Hypertension	−0.11	0.23
Diabetes Mellitus	−0.19	**0.05**
Obesity	−0.14	0.13
Hypercholesterolemia	−0.03	0.75
ACEI/ARB	0.015	0.87
BB	−0.02	0.83
Statin	−0.19	**0.05**
Diuretics	−0.01	0.9
Nitrates	−0.17	0.08
CCB	−0.07	0.48
Phosphate binder	0.002	0.99
K binder	0.08	0.37
erythropoietin	0.12	0.19
NaHCO3	0.03	0.76
NSAIDS	−0.14	0.15
All-cause mortality	0.0036	0.97
Serum Na	−0.17	0.07
PH	−0.16	0.09
NaHCO3	−0.22	**0.02**
PO4	0.25	**0.01**

Univariate analysis for predictors of LVGLS revealed significant association with statin use (*p* = 0.02), while multivariate analysis for predictors of LVGLS revealed no significant association with any clinical or laboratory parameters (Table 4).

**Table 4:** **Predictors of GLS (**≥-**11 %) in patients with CKD with normal ejection fraction by logistic regression analysis**.

Variables	Univariate		Multivariate	
	OR (95 % CI)	*P* value	OR (95 % CI)	*P* value
Age (years)	1.01 (0.98–1.2)	0.38		
Sex	0.65 (0.28–1.51)	0.32		
Hypertension	2.03 (0.72–5.7)	0.16		
Diabetes Mellitus	1.9 (0.86–4.2)	0.10	1.85 (0.53–6.5)	0.33
Obesity	2.42 (0.77–7.5)	0.11	2.21 (0.57–8.5)	0.24
Hypercholesterolemia	0.94 (0.4–2.15)	0.88		
ACEI/ARB	1.19(0.48–2.94)	0.69		
BB	0.78 (0.33–1.83)	0.57		
Statin	2.5 (1.08–5.74)	**0.02**	1.58 (0.50–4.90)	0.42
Diuretics	0.8 (0.35–1.86)	0.61		
CCB	1.27 (0.55–2.90)	0.57		
erythropoietin	0.78 (0.35–1.7)	0.54		
NaHCO3	0.96 (0.4–2.2)	0.92		
egfr (ml/min/1.73m^2^)	0.99 (0.98–1.01)	0.93		
Hb (g/dl)	1.06 (0.89–1.27)	0.47		
Creatinine (mg/dl)	0.96 (0.86–1.08)	0.56		
Urinary ACR (mg/g)	0.99 (0.98–1.00)	0.78		
Serum albumin (g/dl)	1.73 (0.94–3.19)	0.06	1.98 (0.92–4.2)	0.078
Na (mmol/L)	1.05 (0.95–1.15)	0.31		
K (mmol/L)	1.09 (0.68–1.74)	0.7		
Ca (mg/dl)	1.16 (0.73–1.82)	0.51		
PO4 (mg/dl)	0.82 (0.61–1.09)	0.17		
Vit D (ng/dl)	1.008 (0.97–1.04)	0.61		
iPTH (pg/ml)	1.001(0.99–1.002)	0.12		
Mg (mg/dl)	0.94 (0.39–2.25)	0.89		
Ferritin (ng/ml)	0.999(0.998–1.001)	0.91		
HbA1C (%)	1.09 (0.85–1.40)	0.45		
NaHCO_3_ (mEq/L)	1.05 (0.95–1.15)	0.30		
PH	1.36 (0.003–593)	0.91		
Uric acid (mg/dl)	1.03 (0.89–1.2)	0.63		
Total cholesterol (mg/dl)	0.99 (0.98–1.004)	0.42		
TG (mg/dl)	1.0005(0.99–1.006)	0.84		
LDL (mg/dl)	0.99 (0.98–1.01)	0.92		
TSH (mIU/L)	1.04 (0.95–1.14)	0.31		

## 5 Discussion

In our study, most patients (91 %) had CKD stages V, IV, and IIIb. Males were predominant (66 %), which is consistent with the study by Garg et al.^9^ Abnormal GLS (≥-16 %) was prevalent in 98 % of the study population. This is quite a bit higher as compared to previous studies even after excluding diseases like valvular heart disease, ischemic heart disease which affect LVGLS. This prevalence was 32 % in study conducted by Hensen et al,^10^ 58 % in the study by Liu et al,^11^ 89 % in a study by Pressman et al^12^ conducted in 48 African- American CKD patients with preserved LVEF. The much higher prevalence of abnormal LVGLS in our study may be related to lesser use of nephroprotective and cardioprotective drugs e.g., ACEI/ARB 28 %, compared to 65 % in the study by Hensen et al.^10^

Mean LVGLS in our study population was 11 % (±2.74). Patients with LVGLS ≥ -11 % had more comorbidities, a finding that is in concordance with a previous study by Hensen et al.^10^ LVH was present in 61 % of study population, while in the study by Liu et al,^11^ it was 68 %. Variables associated with development of LVH in CKD patients are systemic hypertension, increased volume overload, increased arterial stiffness, anaemia, increased calcium-phosphate metabolites and activation of Renin-angiotensin- aldosterone system.^13^

Pearson's pairwise correlations showed that GLS was negatively correlated with diabetes mellitus (*p* = 0.05), statin requirement (*p* = 0.05), serum sodium level (*p* = 0.07), serum sodium bicarbonate level (*p* = 0.02), PH (*p* = 0.09), while showing a positive correlation with phosphate (*p* = 0.01). In the 1-year median follow-up, all-cause mortality was 22 % (*n* = 21) and was in concordance with previous studies. Among these patients, 60 % were on haemodialysis, and there was no correlation between abnormal GLS and mortality.

## 6 Limitations

This prospective cohort study had several limitations. First, the follow-up period was relatively short. Second, the population size was small. Third, the study was single-centered.

## 7 Conclusion

Patients with abnormal LVGLS (≥-11 %) had more comorbidities. Abnormal global longitudinal strain (GLS > −16 %) was prevalent in 98 % of the study population. The higher prevalence compared to other studies may be related to the lesser use of nephroprotective and cardioprotective drugs (e.g., ACEI/ARB in only 28 %); however, abnormal GLS was not correlated with mortality.

## Funding

None.

**What is already known**- 10.13039/100014821GLS detects early changes in the heart's pumping ability, and is an independent predictor of all cause and cardiovascular mortality in CKD patients.

**What this study adds**- This study reveals prevalence of abnormal GLS and its correlation with mortality in CKD patients in Indian context.

## Declaration of competing interest

The authors declare that they have no known competing financial interests or personal relationships that could have appeared to influence the work reported in this paper.
